# Inhibition of soluble epoxide hydrolase increases coronary perfusion in mice

**DOI:** 10.14814/phy2.12427

**Published:** 2015-06-15

**Authors:** Jun Qin, Dong Sun, Houli Jiang, Sharath Kandhi, Ghezal Froogh, Sung Hee Hwang, Bruce D Hammock, Michael S Wolin, Carl I Thompson, Thomas H Hintze, An Huang

**Affiliations:** 1 Department of Physiology, New York Medical CollegeValhalla, New York; 2 Department of GI Surgery, Renji Hospital, Shanghai Jiaotong University, School of MedicineShanghai, China; 3 Department of Pharmacology, New York Medical CollegeValhalla, New York; 4 Department of Entomology, University of California Davis Comprehensive Cancer Center, University of CaliforniaDavis, California

**Keywords:** Cardiac work, coronary circulation, epoxyeicosatrienoic acids, soluble epoxide hydrolase

## Abstract

Roles of soluble epoxide hydrolase (sEH), the enzyme responsible for hydrolysis of epoxyeicosatrienoic acids (EETs) to their diols (DHETs), in the coronary circulation and cardiac function remain unknown. We tested the hypothesis that compromising EET hydrolysis/degradation, via sEH deficiency, lowers the coronary resistance to promote cardiac perfusion and function. Hearts were isolated from wild type (WT), sEH knockout (KO) mice and WT mice chronically treated with *t*-TUCB (sEH inhibitor), and perfused with constant flow at different pre-loads. Compared to WT controls, sEH-deficient hearts required significantly greater basal coronary flow to maintain the perfusion pressure at 100 mmHg and exhibited a greater reduction in vascular resistance during tension-induced heart work, implying a better coronary perfusion during cardiac performance. Cardiac contractility, characterized by developed tension in response to changes in preload, was potentially increased in sEH-KO hearts, manifested by an enlarged magnitude at each step-wise increase in end-diastolic to peak-systolic tension. 14,15-EEZE (EET antagonist) prevented the adaptation of coronary circulation in sEH null hearts whereas responses in WT hearts were sensitive to the inhibition of NO. Cardiac expression of EET synthases (CYP2J2/2C29) was comparable in both genotypic mice whereas, levels of 14,15-, 11,12- and 8,9-EETs were significantly higher in sEH-KO hearts, accompanied with lower levels of DHETs. In conclusion, the elevation of cardiac EETs, as a function of sEH deficiency, plays key roles in the adaptation of coronary flow and cardiac function.

## Introduction

Following the acceptance of cyclooxygenase (COX) and lipoxygenase metabolites of arachidonic acid (AA) as clinical targets for the treatment of inflammatory disorders, the cytochrome P-450 (CYP/epoxygenase) enzyme has emerged as another noteworthy pathway for AA metabolism. AA metabolism by CYP/epoxygenase results in the production of epoxyeicosatrienoic acids (EETs), metabolites that have intrigued researchers and gained considerable attention due to their bioactive properties, which have been shown to be involved in cellular signaling and cardiovascular protection (Lee et al. 2010b; Harris and Hammock 2013). Correspondingly, soluble epoxide hydrolase (sEH) functions by catalyzing the conversion of EETs to their corresponding diols (DHETs) that are either less active, or lacking in bioactivity all together (Newman et al. 2005). A review of the literature shows that potentiation of EET bioavailability through compromising EET degradation via either knockout of the Ephx2 gene that encodes the sEH enzyme, or by pharmacological inhibition of the enzyme, evokes cardiovascular protective actions (Imig 2006; Imig and Hammock 2009). During the process of sEH-specific modulation of cardiovascular function, compelling evidence confirms the sEH deficiency-dependent prevention of hypertension in varying pathologies (Chaudhary et al. 2013b; Goto et al. 2013). On the other hand, upregulation of sEH associated with a marked increase in EET hydrolysis in some spontaneous hypertensive rats (SHR) is recognized as an etiology of their spontaneous hypertension (Yu et al. 2000). Additionally, increased sEH mRNA was reported to be responsible for the development of hyperglycemic stroke and cerebral infarction in type I and type II diabetic models (Jouihan et al. 2013; Zuloaga et al. 2014). The deletion of the Ephx2 gene- or inhibition of the enzyme-initiated cardio-protective actions have also been widely evidenced in a variety of pathological conditions such as, neuronal and cardiac ischemia (Koerner et al. 2007; Lee et al. 2010b); hypertension-induced renal injury (Lee et al. 2010a); and cardiac damage induced by ischemia–reperfusion injury (Wu et al. 1996; Chaudhary et al. 2013a,b). It is worth noting that pathological models, such as in the aforementioned studies, have been consistently used throughout sEH-related research, especially work by Seubert et al., a group that has used Langendorff perfusion methods and have provided strong evidence for protection against ischemia/reperfusion induced cardiac injury in sEH-deficient models (Chaudhary et al. 2009; Batchu et al. 2012a,b). To date, however, fewer studies have been carried out to specifically clarify the physiologically based significance of sEH inhibition in the regulation of cardiac function. In a recently published study, we demonstrated that under the normal physiological state, deletion of the Ephx2 gene or pharmacological inhibition of sEH activity attenuates myogenic constriction in skeletal muscle arterioles, leading to an attenuation in vascular tone and consequently, reduced blood pressure (Sun et al. 2014). In this study, we have extended and updated our experimental preparation from isolated single arteries to isolated intact hearts, aimed at an intact circulation, to elucidate whether the reduction in arteriolar constriction benefits cardiac blood supply, followed by potentiation of cardiac performance. The Langendorff preparation is a fundamental approach and believed to be the most extensively used isolated organ model to investigate a myriad of scientific questions on heart biology; from the effect of a single gene alteration on heart physiology to therapeutic means of protecting the heart from ischemia and other insults (Skrzypiec-Spring et al. 2007). Given that the physiological relevance of an altered/absent Ephx2 gene in the regulation of coronary circulation is currently unknown, we tested the hypothesis that in physiological conditions increases in cardiac EETs, as a function of diminishing their degradation, potentiates coronary flow and improves cardiac function. By using Langendorff-perfused hearts isolated from sEH-KO mice, and normal mice receiving a pharmacological inhibitor of sEH for four weeks, we provide evidence for EET-mediated adaptation of the coronary circulation and potentiation of cardiac performance via the mechanism of promoting coronary blood supply and attenuating vascular resistance to improve cardiac perfusion.

## Materials and Methods

### Animals

Twelve- to fifteen-week-old male Ephx2^−/−^ (sEH-KO) and Ephx2^+/+^ (wild type, WT) mice were used. As described previously (Sun et al. 2014), cryorecovered heterozygoues (Ephx2^+/−^, B6.129X-Ephx2tm1Gonz/J) and WT mice were received from the Jackson laboratory (Bar Harbor, ME) and the homozygous (sEH-KO) mice were developed in the Department of Comparative Medicine, New York Medical College. One group of WT mice received *t*-TUCB (a sEH inhibitor, 1 mg/kg/day), via oral gavage, for 4 weeks.

All protocols were approved by the Institutional Animal Care and Use Committee of New York Medical College and conform to the guidelines of the National Institutes of Health and the American Physiological Society for the use and care of laboratory animals.

### Measurement of blood pressure

Mice were anesthetized by inhalation of Isothesia (isoflurane). Blood pressure was recorded using carotid artery catheterization and a flow-through pressure transducer (TRN050; Kent Scientific, Torrington, CT). The heart rate of anesthetized mice was controlled at approximately 480 beats per minute by adjusting the depth of anesthesia when the blood pressure was measured. In *t*-TUCB-treated mice, changes in blood pressure were monitored twice per week by tail-cuff blood pressure measurements during the course of treatment, as well as by carotid artery catheterization on the day the experiment was conducted.

### Isolated-perfused hearts

As described previously (Tada et al. 2000) mice were anesthetized with isoflurane, and heparin (100 U) was injected intraperitoneally. The thorax was then opened. The heart was excised and immediately mounted via the ascending aorta onto a perfusion apparatus. The heart was perfused with a nonrecirculating PSS containing (in mmol/L) NaCl 117, KCl 4.7, MgSO_4_ 1.1, KH_2_PO_4_ 1.2, glucose 5.5, CaCl_2_ 2.5, pyruvate 2.0, ascorbate 0.1, L-arginine 0.1, and NaHCO_3_ 24. The PSS was gassed in a water-jacketed (37°C) reservoir with 95%O_2_–5% CO_2_ to a pH value of 7.4. The perfusate was pumped through an in-line 1 *μ*m filter using a Rainin peristaltic pump. A 2-mL bubble trap, a water-jacketed (37°C) heating coil and a T-adaptor connecting a pressure transducer (P23XL, Grass, Quincy, MA) were sequentially connected to the heart. A metal hook made from a 31 gauge needle was penetrated through the left ventricle at the apex of the heart to drain the Thebesian fluid accumulated in the ventricle. The other end of the hook was attached to a force transducer (FTO3C; Grass) to control and record tension and heart rate. The heart was immersed in the coronary effluent solution in a water-jacketed (37°C) tissue bath (10 mL; Radnoti, Monrovia, CA). The heart was perfused with a constant flow. Changes in perfusion pressure, end-diastolic, and peak-systolic tension were continuously recorded. At the beginning of experiments, the flow rate was rapidly increased to reach a perfusion pressure of 100 mmHg. During the equilibration, changes (increases) in perfusion pressure as a consequence of development of myogenic constriction in coronary arteries were continuously adjusted by reducing flow rate to maintain a perfusate pressure at 100 mmHg. The hearts reached an equilibrated status (a constant perfusion pressure of 100 mmHg and a constant heart rate of above 320 beats per min) within 15 min. The flow rate provided to obtain a constant perfusion pressure of 100 mmHg was considered as basal coronary flow and was kept constant throughout experiments. After basal flow was obtained, tension was increased from 0 to 5 g in 0.5-g increments to initiate a step-wise increase in end-diastolic tension of the heart. At each tension step, changes in perfusion pressure were continuously recorded for 5 min on a DI-700 data acquisition system (DATAQ Instruments, Akron, OH).

In separate groups of mice, N^*ω*^-nitro-L-arginine methyl ester (L-NAME, 3 × 10^−4^mol/L; NOS inhibitor) or 14,15-epoxyeicosa-5(Z)-enoic acid (14,15-EEZE, 10^−5^mol/L; a putative pan-EET receptor antagonist), or L-NAME plus EEZE were added into the PSS. Hearts were perfused with inhibitor-containing PSS for 45 min before recording the basal flow, and then changes in perfusion pressure in responses to stepwise-increases in tension were recorded.

### Heart weight measurement

After perfusion experiments, hearts were dabbed on paper towel to remove excessive fluid and weighted to obtain the wet weight of hearts. Hearts were then wrapped with aluminum foil and placed in an 80°C oven for at least 48 h until a constant dried weight obtained.

### LC/MS/MS-based measurements for cardiac EETs and DHETs

Isolated hearts were pulverized in liquid nitrogen. Phospholipids were extracted with the Bligh-Dyer method from 30 to 50 mg of heart tissue. EETs and DHETs were extracted with ethyl acetate following alkali hydrolysis of the phospholipids to release esterified EETs or DHETs and quantified with a Q-trap 3200 linear ion trap quadruple LC/MS/MS (AB ScieX; Qtrap 3200, Framingham, MA) equipped with a Turbo V ion source operated in negative electrospray mode (Applied Biosystems, Foster City, CA), as described previously (Inoue et al. 2009; Sun et al. 2014). Protein concentration of samples was determined by the Bradford method (Bio-Rad, Hercules, CA) and was used to normalize the detected lipids. Data are presented as each of four regioisomeric EETs and DHETs and expressed as pg/mg protein.

### Western blot analysis

Isolated hearts were pulverized in liquid N_2_. Equal amounts of total protein (25 *μ*g) extracted from hearts were loaded on and separated by a 10% SDS-PAGE gel and transferred to a PVDF membrane. The membrane was probed with specific primary antibodies for sEH, eNOS, CYP2J2 (Santa Cruz Biotechnology Inc., Santa Cruz, CA), and CYP2C29 (Biodesign Inc., Maco, ME), respectively, and appropriate secondary antibodies conjugated with horseradish peroxidase. Specific bands were visualized with a chemiluminescence kit and normalized to GAPDH. The X-ray film was scanned into a computer and band densitometry was digitalized with UN-SCAN-IT software.

### Calculation and statistical analysis

Data are expressed as means ± SEM and n refers to the number of mice. Basal coronary flow was recorded at a constant pressure of 100 mmHg and normalized to heart weight, and expressed as mL/min/g. Coronary vascular resistance was calculated by the formula of R = P/F, where P and F are the indicative of perfusion pressure and flow, respectively, and expressed as mmHg/min/mL. Analyses of linear regression and the comparison of the slopes of regression lines between multiple groups were performed using GraphPad Prism software (GraphPad Software, La Jolla, CA). Analyses of animal and heart parameters, coronary basal flow and cardiac level of metabolites were performed using Student's *t*-test or pair *t*-test where appropriate. Statistical significance was accepted at a level of *P* < 0.05.

## Results

Table1 provides general information of WT and sEH-KO mice, as well as WT mice treated with *t*-TUCB for 4 weeks. Blood pressure including systolic, diastolic, and mean arterial pressure, was significantly reduced in mice with the sEH gene genetically knocked down or sEH activity pharmacologically inhibited as compared to their respective WT controls. Body weight (BW) was comparable among the three groups, but heart weight (HW; both in wet and dry conditions) was significantly increased in sEH-KO compared to WT mice, leading to a greater ratio of HW/BW in sEH-KO mice. The ratio of HW/BW between *t*-TUCB-treated WT and WT controls was comparable.

**Table 1:** Characters of mice

	WT (*n* = 12)	sEH-KO (*n* = 9)	WT with *t*-TUCB (*n* = 6)
SBP (mmHg)	116 ± 3	104 ± 2†	105 ± 2†
DBP(mmHg)	90 ± 4	72 ± 3†	79 ± 4†
MAP (mmHg)	98 ± 3	83 ± 3†	88 ± 4†
HR (beats per min)	479 ± 16	485 ± 16	480 ± 21
BW (g)	23.7 ± 0.7	23.8 ± 1.3	26.4 ± 0.4
DHW (mg)	21 ± 1.2	25 ± 4.8†	24.1 ± 8.7
HW/BW (mg/g)	0.88 ± 0.06	1.05 ± 0.17†	0.91 ± 0.23

SBP, systolic blood pressure

DBP, diastolic blood pressure

MAP, mean arterial pressure

HR, heart rate

BW, body weight

DHW, dry heart weight

HW/BW, ratio of dry heart weight over body weight.

† Significant difference from WT controls.

### Myocardial EETs

The functional consequence of Ephx2 gene deletion was documented by LC/MS/MS analysis. Table2 shows that cardiac levels of 14,15-EET, 11,12-EET, and 8.9-EET were significantly elevated, paralleled with reductions in 14,15- and 11,12-DHETs to enhance their EET/DHET ratios in sEH-KO mice. 5,6-EET and 5,6-DHET were comparable between sEH-KO and WT mice; suggesting that 5,6-EET is a poor substrate for sEH (Zeldin et al. 1995).

**Table 2:** Myocardial cytochrome P450 metabolites

	WT (*n* = 7)			sEH-KO (*n* = 6)		
	EET	DHET	EET/DHET	EET	DHET	EET/DHET
14,15-	1.413 ± 0.324	0.063 ± 0.021	54.4 ± 27.3	2.438 ± 0.279†	0.022 ± 0.007†	149.915 ± 29.22†
11,12-	1.736 ± 0.351	0.049 ± 0.024	180.82 ± 80.00	4.034 ± 0.603†	0.034 ± 0.018†	255.341 ± 59.6†
8,9-	2.226 ± 0.379	0.113 ± 0.012	20.695 ± 3.031	5.379 ± 1.503†	0.135 ± 0.024	56.294 ± 23.055
5,6-	2.414 ± 0.480	0.229 ± 0.071	18.565 ± 7.919	4.300 ± 0.611	0.457 ± 0.117	14.042 ± 5.007

EETs and DHETs are quantified as picogram per milligram of tissue.

† Significant difference compared to corresponding value in WT mice.

### sEH deficiency increases coronary flow

**Figure 1:**
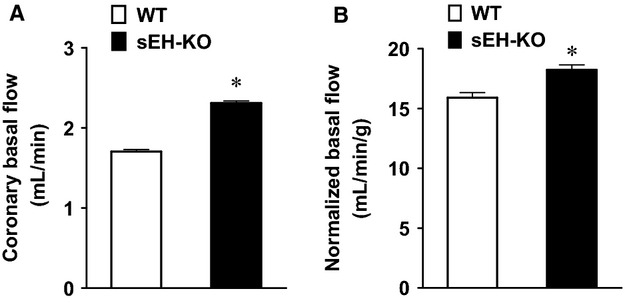
Coronary basal flow was obtained at zero preload (A) and then was normalized to the heart weight (B) in WT (*n* = 12) and sEH-KO (*n* = 9) mice. *indicates a significant difference from WT controls.

Figure1 summarizes the data of basal coronary flow in both strains of mice, showing that in order to maintain a constant perfusion pressure of 100 mmHg, a significantly greater basal coronary flow was required in sEH-KO mice compared to that in WT controls (Fig.1A). The increase remained significant after the data were normalized to the heart weight (Fig.1B), suggesting that sEH deficiency promotes coronary perfusion.

**Figure 2:**
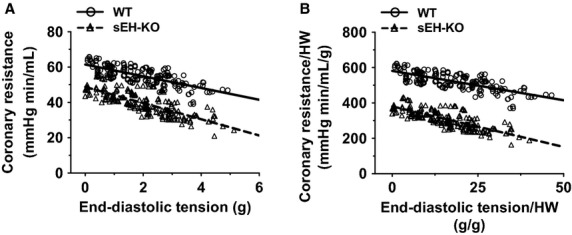
The correlation between coronary vascular resistance and end-diastolic tension in isolated hearts of WT (*n* = 12) and sEH-KO (*n* = 9) mice. (A) Correlation coefficient is −0.7316 (*P* < 0.0001) and −0.7702 (*P* < 0.0001) in WT and sEH-KO hearts, respectively. The slope of regression line is -3.329 ± 0.2358 (WT) and −4.582 ± 0.271 (sEH-KO). The difference between the two slopes is statistically significant (*P* < 0.001). (B) Changes in end-diastolic tension and coronary resistance were normalized by heart weights. Correlation coefficient is −0.7348 (*P* < 0.0001) and −0.7715 (*P* < 0.0001) in WT and sEH-KO hearts, respectively. The slope of regression line is −3.322 ± 0.2346 (WT) and −4.593 ± 0.2722 (sEH-KO). The difference between the two slopes is statistically significant (*P* < 0.0001).

Next, changes in perfusion pressure expressed as changes in vascular resistance, were recorded in response to step-wise increases in end-diastolic tension in hearts of WT and sEH-KO mice. As depicted in Fig.2, coronary resistance at zero diastolic tension was inversely proportional to the level of basal perfusion flow shown in Fig.1. Increases in end-diastolic tension dose-dependently reduced vascular resistance in both genotypes of hearts, but the magnitude of reduction at each preload tested, was greater in sEH-KO than that of WT hearts, which is exhibited by a statistically significant difference between the slopes of their respective regression lines. This revealed that coronary arteries of sEH-KO mice exhibit greater dilator responses to the increases in cardiac work compared to WT. We also normalized the data (Fig.2A) to the heart weight and are depicted in Fig.2B. We found that the normalized data (Fig.2B) shows the same pattern in regression lines, as well as similar slopes as compared to data not normalized to heart weight (panel A). These results suggest that the significant reduction in coronary resistance of sEH-KO heart, compared to WT, is independent upon its cardiac hypertrophy. Also, we noticed that the normalization changes the absolute tension (*x*-axis) received by the heart to a group of relative numbers. Given that the normalization does not alter the statistical significance, but has an unnecessary impact on the expression of data, the analysis of the data without normalization is therefore, preferred over the normalized data.

### sEH deficiency increases cardiac contraction

**Figure 3:**
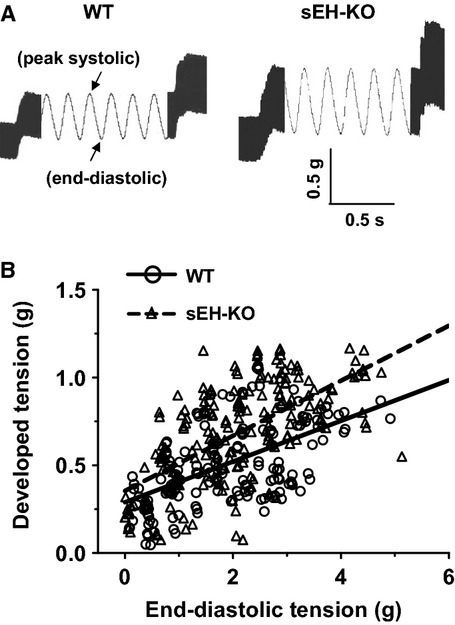
(A) Representative tracings of heart contraction (peak-systolic tension) in response to 0.5 g end-diastolic tension in WT and sEH-KO mice. (B) Linear relationships between developed tension (difference of peak-systolic tension and end-diastolic tension) and end-diastolic tension in both stains of mice. Correlation coefficient equals 0.5541 (*P* < 0.0001) and 0.6426 (*P* < 0.0001) in WT (*n* = 12) and sEH-KO (*n* = 9) hearts, respectively. The slopes of two regression lines are significantly different from each other (*P* < 0.05).

Developed tension, represented as a difference between peak-systolic and end-diastolic tension, was compared between WT and sEH-KO hearts in response to increases in end-diastolic tension. As shown in Fig.3, both groups of hearts exhibited dose-dependent increases in the magnitude of developed tension. Moreover, in sEH-KO hearts, the increment at each point of preload was significantly greater than that of WT hearts, as indicated by a deeper slope of the regression line.

### EET contributes to the enhanced coronary responses

**Figure 4:**
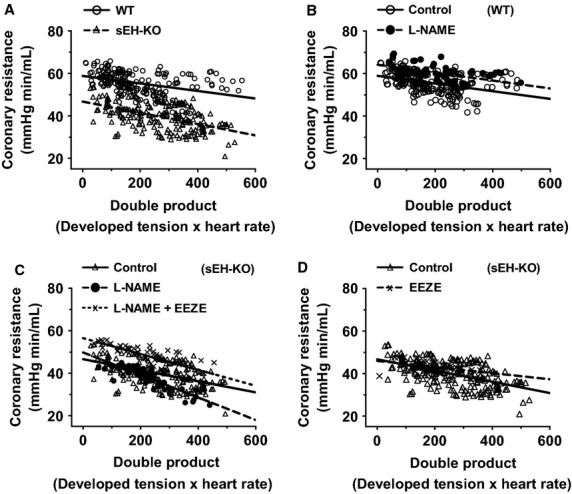
Linear relationships between coronary vascular resistance and double product. (A): Correlation coefficient equals −0.3908 (*P* < 0.0001) and −0.5262 (*P* < 0.0001) in WT (*n* = 12) and sEH-KO (*n* = 9) hearts, respectively. The slopes of two regression lines (WT: −0.01773 ± 0.002954 vs. sEH-KO: −0.02647 ± 0.002987) are statistically different (*P* < 0.05). (B) WT: Correlation coefficient equals −0.3779 (*P* < 0.0001) and −0. 5548 (*P* < 0.0001) in WT control (*n* = 12) and in the presence of L-NAME (*n* = 5) hearts, respectively. The two slopes of regression lines (Control: −0.01808 ± 0.003172; L-NAME: −0.01867 ± 0.003473) are comparable (*P* = 0.9144), whereas, L-NAME induced increment of coronary resistance is statistically significant compared to their controls (*P* < 0.0001). (C) sEH-KO: Correlation coefficient equals −0.5022 (*P* < 0.0001), −0.8689 (*P* < 0.0001) and −0.742 (*P* < 0.0001) in the control (*n* = 9), L-NAME (*n* = 6) and L-NAME+EEZE (*n* = 5) hearts, respectively. The slope of regression line in the presence of L-NAME+EEZE (−0.03737 ± 0.004682) is not different (*P* = 0.1296) from that of controls, but the increment of coronary resistance in the presence of the two inhibitors is statistically significant from their controls (*P* < 0.0001). (D) sEH-KO: Correlation coefficient equals −0.5262 (*P* < 0.0001) and −0.4413 (*P* < 0.0004) in control hearts (*n* = 12) and hearts in the presence of EEZE (*n* = 5), respectively. The two slopes of regression lines (Control: −0.02647 ± 0.002987; EEZE: −0.01449 ± 0.003836) are comparable (*P* = 0.211), but the increment of coronary resistance by EEZE is statistically significant from their controls (*P* = 0.0291).

On the basis of aforementioned findings, we next aimed to elucidate underlying mechanisms responsible for the enhanced coronary flow and cardiac contraction in sEH-KO mice. The correlation of cardiac work, which was quantitated by the double product (developed tension × heart rate), and coronary resistance was compared between the two groups. Figure4A shows that with increases in cardiac work, coronary resistance was gradually reduced in both groups of hearts, whereas the rate of reduction was significantly greater in sEH-KO than those in WT mice, suggesting an enhanced vasodilator response in the coronary arteries of sEH-KO mice. To determine the contribution of NO and EETs in the control of coronary responses, L-NAME and 14,15-EEZE were used. As shown in Fig.4B, when subjecting WT hearts to L-NAME, the correlation of cardiac work and coronary perfusion remains, but the regression line is shifted upward significantly, indicating increases in coronary resistance in response to L-NAME. This result confirms a major contribution of NO to the control of coronary resistance in WT mice. In sEH-KO mice (Fig.4C), however, L-NAME did not inhibit the reduction in vascular resistance in response to the increases in cardiac work, but rather elicited a further reduction of resistance, evidenced by a downward shift of the linear regression line. The predominant reduction of vascular resistance in sEH-KO hearts in response to L-NAME was then, prevented by additional administration of 14,15-EEZE. Moreover, EEZE alone that had no effect on WT hearts (data not shown), significantly elevated the coronary resistance in sEH-KO hearts (Fig.4D), confirming further a major contribution of EETs to the regulation of coronary resistance, as a function of absent sEH. Thus, this analysis reveals two regulatory mechanisms: (1) adaptation of coronary circulation is mediated by myocardial EETs; and (2) there is an inhibitory effect of NO on the activity of EETs.

### Western blot analysis

**Figure 5:**
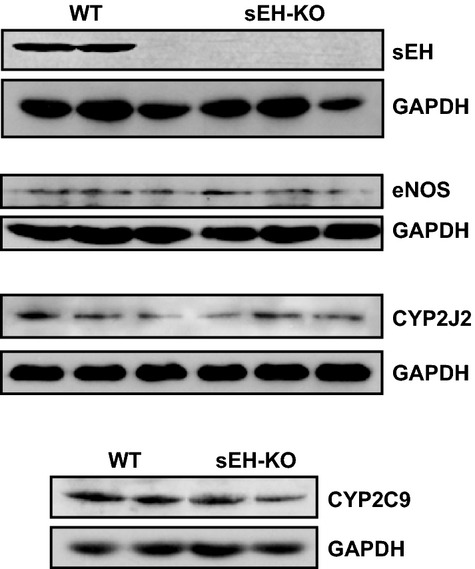
Protein expression of sEH, eNOS CYP2J2, and CYP2C9 in hearts of WT and sEH-KO mice.

Figure5 shows that protein expression of EET synthases including CYP2J2 and CYP2C29 in myocardial tissues was comparable in both strains of mice. Also, there was no difference in the expression of eNOS between WT and sEH-KO mice.

### Pharmacological inhibition of sEH

**Figure 6:**
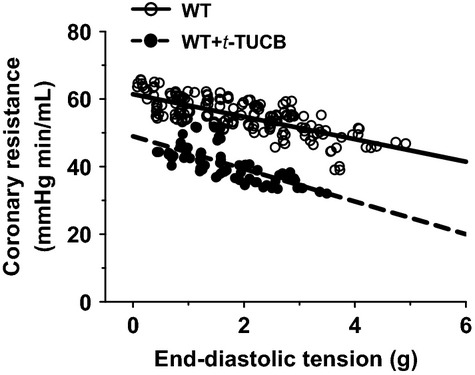
The relationship between coronary vascular resistance and end-diastolic tension in hearts of WT (*n* = 12) and WT-treated with *t*-TUCB (1 mg/kg/day, *n* = 5) for 4 weeks. In WT controls, correlation coefficient is −0.7316 (*P* < 0.0001) and the slope of regression line is −3.329 ± 0.2358. In WT-treated with t-TUCB mice, correlation coefficient is −0.7177 (*P* < 0.0001) and the slope of regression line is −4.833 ± 0.5319. The two slopes are significantly different (*P* < 0.001).

In separate experiments, sEH-dependent modulation of the coronary circulation was evaluated in WT mice that had been treated with *t*-TUCB (an inhibitor of sEH) for 4 weeks. Figure6 shows that treatment of WT mice with *t*-TUCB resulted in a downward-shifted regression line in the end-diastolic vs. resistance curve. The rate of reduction in coronary resistance was significantly greater in *t*-TUCB-treated than that of control mice, as evidenced by a deeper slope.

## Discussion

This study demonstrates the specific role of deleting the sEH gene or pharmacological inhibition of sEH enzyme in the adaptation of coronary circulation, characterized as enhanced coronary flow (Fig.1) and attenuated vascular resistance (Fig.2), leading to augmented cardiac performance (Fig.3), associated with a significant reduction in blood pressure (Table1). Consistent with our previous findings (Sun et al. 2014), drastic increases in cardiac EETs with subsequent decreases in DHETs (Table2) are functionally indicative of a reduced capacity for EET hydrolysis.

### Deficiency of sEH improves coronary circulation

Evidence of EET-dependent protection of cardiac function, characterized by protection against cardiac damage, was mainly obtained from studies conducted on postischemic recovery or ischemia/reperfusion models of sEH null hearts (Chaudhary et al. 2009, 2013a,b), or hearts undergoing cardiac hypertrophy (Aboutabl et al. 2011; Althurwi et al. 2013), heart failure (Qiu et al. 2011; Xu et al. 2013), and myocardial infarction (Xu et al. 2013; Shrestha et al. 2014), in response to treatment with sEH inhibitors. To date, fewer studies have reported on the impact of sEH deficiency on physiological function aspects of the coronary circulation. In this regard, we aimed to evaluate the specific regulation of coronary flow and resistance, as well as cardiac contractility under both basal conditions and during cardiac performance in hearts that are deficient for functional sEH. We demonstrated that in physiological conditions, compromised EET degradation is capable of initiating an increase in basal coronary flow while promoting a prominent decline in vascular resistance during cardiac work (Figs.1, 2 and 6). It is important to note that the basal coronary flow we recorded in WT hearts (∽1.7 mL/min) was lower than that reported by others (Kriska et al. 2013). This is possibly due to the fact that in comparison with the natural heart rate of ∽320 beat/min in our studies, the referenced studies of others used pacing hearts in their research, which led to a corresponding heart rate of 420 beats/min (Kriska et al. 2013), thereby exerting a greater blood supply to meet the higher metabolic demand of the heart. In order to address potential concerns related to the presence of any mechanistically based discrepancies caused by transgenic deletion of the Ephx2 gene (sEH-KO) or pharmacological interventions (sEH inhibitors), the same protocols were performed in hearts of WT mice that had chronically received *t*-TUCB. Both groups of mice exhibit a similar responsive phenotype (Fig.6 vs. Fig.2), along with an identical pattern of changes in blood pressure (Table1). This reveals a universal outcome in the regulation of coronary circulation and control of blood pressure, as a function of sEH deficiency, regardless if the deficiency is genomically or pharmacologically based. To the best of our knowledge, this is the first study elucidating a physiologically based EET-dependent adaptation of coronary circulation in normal hearts. This study can serve as a fundamental basis for mechanistically explaining the findings that show improved cardiovascular function in sEH-deficient disease models.

### Adaptation of coronary circulation benefits cardiac function

Cardiac function was specifically characterized as a development of cardiac tension in response to stretched tension, which reflects cardiac contractility. An enhanced contractile capacity in hearts from sEH-KO mice was indicated by an enlarged magnitude of the end-diastolic to peak-systolic tension (Fig.3A). This demonstrates that during the process of cardiac performance, sEH null hearts exhibited a greater potential for tension development, which could be attributed to optimal utilization of oxygen as a consequence of better tissue perfusion. We also noticed that in ischemia/reperfusion injury models, sEH null hearts exhibited much less cardiac damage and better functional recovery in comparison with WT hearts, but the basal contractility of both phenotypic hearts was reported to be comparable in the control/preischemic condition (Chaudhary et al. 2009, 2013a,b). This confounding finding may result from the different parameters used for the evaluation of cardiac contractility (developed tension verses LVDP), and moreover could suggest that the parameter of developed tension used in this study can serve as a sensitive index of cardiac contractility. Although heart-stretch is unable to completely simulate *in vivo* cardiac preload, the developed tension in response to the stretch certainly represents the contractile capacity of intact hearts. Also, we used double product of hearts as a parameter (Fig.4) to normalize any alterations induced by individual variance in heart rate, aimed to exclude the possibility that the potential cardiac work was caused by increases in heart rate. As expected, the same double product of hearts continuously evoked greater vasodilatations in sEH-KO than WT mice (Fig.4A), confirming further that the response is independent of differences in heart rate. Inhibition of NO synthesis blunted cardiac work-induced reduction of vascular resistance in WT mice (Fig.4B), while promoting EET-dependent adaptation in sEH-KO hearts (Fig.4C). In this regard, we interpreted our findings to mean that there is a negative interaction between NO and EETs, such that inhibition of NO synthesis releases the inhibitory effect of NO on EET activity. The phenomenon of negative feedback inhibition of EETs by NO has previously been reported, and indicates that EET activity and its contribution to the regulation of vascular function are dampened under physiological conditions, and become discernable in most instances, only with decreased endothelial NO synthesis or bioavailability (Huang et al. 2000, 2001, 2005; Wu et al. 2001). Indeed, 14,15-EEZE initiated significant increases in coronary resistance of sEH-KO hearts (Fig.4D), but the magnitude of increment was much smaller than that observed in the additional presence of L-NAME (Fig.4C), further revealing the interaction between these two mediators. Collectively, 14,15-EEZE prevented the adaptation in sEH null hearts without significant effects on WT responses, providing solid evidence of an EET-dependent response (Fig.4C and D).

### Cardioprotective mechanisms

Increased cardiac EETs activate various pathways for signaling cardio-protective responses. In our studies, the enhanced EET-dependent vasodilation and improved cardiac perfusion may play major roles, presumably accompanied with a promotion of cardiac substrate uptake, in the adaptation of coronary circulation and enhancement of cardiac function in sEH-KO mice. In physiological conditions, the heart derives most of the energy necessary for its contractile function from fatty acid oxidation under aerobic conditions (Lopaschuk et al. 1994). In this context, the type of oxidized substrate may influence the efficiency of normal hearts. Previous studies have also demonstrated that within the signaling network of fatty acid metabolism via CYP/epoxygenase/sEH, peroxisome proliferator-activated receptors (PPARs; transcriptional factors) function as targets of EETs (Imig et al. 2005). As demonstrated, EETs are activators of PPARs (Ng et al. 2007), such that when activated, PPARs are able to increase fatty acid uptake in the heart (Goto et al. 2013). Given that fatty acids serve as a primary energy source for cardiac contractile function (Recchia et al. 1998; Tada et al. 2000), and that sEH appears primarily responsible for the hydroxylation/degradation of epoxy fatty acids such as EETs (Wagner et al. 2014), it is plausible to speculate that with compromised EET hydrolysis, cardiomyocytes are able to optimally utilize EETs as an energy substrate, while the subsequent EET-dependent activation of PPARs further causes vigorous uptake of fatty acids by the myocardium. As such, a positive feed-back cycle forms to support ATP synthesis and promote cardiac performance. Correspondingly, a published study regarding fatty acid metabolism in the heart provided evidence for this speculation, indicating that in mouse cardiomyocytes AA serves as a major fatty acid substrate for CYP2J2, the highest expressed CYP epoxygenase in cardiomyocytes (Seubert et al. 2004).

In addition to the initial identification of EETs as vasodilators, the concept of EET-mediated angiogenesis has been well accepted since it was first reported in 2000 (Munzenmaier and Harder 2000). In pulmonary and coronary vasculatures, EETs initiate activation of angiogenic signaling by promoting endothelial cell proliferation (Medhora et al. 2003; Michaelis et al. 2005) and inhibiting several apoptotic signaling events (Samokhvalov et al. 2013; Chen et al. 2014). Moreover, EET-dependent acceleration of in vivo tissue/organ growth was also demonstrated in the angiogenic process (Panigrahy et al. 2013). As such, while we did not provide direct evidence for the presence of angiogenesis in sEH null hearts, we speculate that the increased heart weight (Table1) and enhanced perfusion flow may refer, at least in part, to the presence of cardiac angiogenesis in this genotypic model.

Hydrolysis of EETs by sEH is tissue-specific, and also occurs in a region- and stereo-selective manner (Zeldin et al. 1995). As demonstrated, CYP2J/2C catalyzes the oxidation of AA at any of the four double bonds to form four correspondingly regioisomeric EETs (Imig and Hammock 2009). On the other hand, CYP2J and 2C are the predominantly expressed CYP epoxygenases favored for metabolizing AA to 14,15- and 11,12-EETs in mouse cardiomyocytes and vascular endothelium (Wu et al. 1996; Sun et al. 2010). LC/MS/MS data (Table2) profiles an altered epoxide:diol ratio for 14,15- and 11,12-EET regioisomers, but not for 5,6-EET regioisomer in hearts of sEH-KO mice, consistent with the known substrate preferences of sEH (Zeldin et al. 1995). In regard to the significant increase in 8,9-EET in sEH-KO hearts, which is paradoxically associated with maintained levels of 8,9-DHET, one possible deduction could be that the DHET levels measured in this study are the portion of DHETs that are incorporated in cellular phospholipids. As 8,9-DHET is more hydrophobic than 14,15- and 11,12-DHET, it is more readily retained in phospholipids. Also, the levels of DHETs in phospholipids are only about 5% that of EETs overall, thus a significant reduction in 8,9-DHET levels may not be easily detected.

In conclusion, the deletion of the Ephx2 gene or inhibition of sEH activity improves the basal coronary circulation via enhanced coronary flow and reduced vascular resistance, leading to potentiation of cardiac performance in normal hearts. This study significantly enhances our understanding of the physiological regulation of the coronary circulation, as a function of sEH inhibition. Moreover, this study highlights a physiological significance of sEH in EET-dependent cardioprotective effects, in terms of utilizing sEH inhibitors as not only therapeutic interventions to protect against cardiac diseases, but also as preventative strategies for the healthy public.

### Alternatives

The rationale for presenting the most of results with original data, instead of those normalized to heart weights, has been briefly, discussed. We found that certain normalizing methods lead to the omission of important information that is critical in understanding the key findings in this study; for instance, the change in slope is an important indicator for the dynamic expression of reactivity of intact hearts in response to stimuli, which could be concealed by the normalization (data not shown). Essentially, regardless of the type of normalization used, the statistical significance between the two strains of hearts was constantly maintained. Also, our aim in using Langendorff-perfused heart was to observe and evaluate cardiac function comprehensively as a whole, via an isolated organ. Therefore, on the basis of these concerns, we have presented most of our data in this study without normalization.

## Conflict of Interest

SHH and BDH are the authors of UC Patents on SEHI chemistry and blood pressure regulation.
